# A Fuzzy-Based Context-Aware Misbehavior Detecting Scheme for Detecting Rogue Nodes in Vehicular Ad Hoc Network

**DOI:** 10.3390/s22072810

**Published:** 2022-04-06

**Authors:** Fuad A. Ghaleb, Faisal Saeed, Eman H. Alkhammash, Norah Saleh Alghamdi, Bander Ali Saleh Al-rimy

**Affiliations:** 1 School of Computing, Faculty of Engineering, Universiti Teknologi Malaysia, Johor Bahru 81310, Malaysia; abdulgaleel@utm.my (F.A.G.); bander@utm.my (B.A.S.A.-r.); 2 College of Computer Science and Engineering, Taibah University, Medina 42353, Saudi Arabia; fsaeed@taibahu.edu.sa; 3 DAAI Research Group, Department of Computing and Data Science, School of Computing and Digital Technology, Birmingham City University, Birmingham B4 7XG, UK; 4 Department of Computer Science, College of Computers and Information Technology, Taif University, P.O. Box 11099, Taif 21944, Saudi Arabia; eman.kms@tu.edu.sa; 5 Department of Computer Sciences, College of Computer and Information Sciences, Princess Nourah Bint Abdulrahman University, P.O. Box 84428, Riyadh 11671, Saudi Arabia

**Keywords:** misbehavior detection, VANET, context-aware, fuzzy inference system, context uncertainty

## Abstract

A vehicular ad hoc network (VANET) is an emerging technology that improves road safety, traffic efficiency, and passenger comfort. VANETs’ applications rely on co-operativeness among vehicles by periodically sharing their context information, such as position speed and acceleration, among others, at a high rate due to high vehicles mobility. However, rogue nodes, which exploit the co-operativeness feature and share false messages, can disrupt the fundamental operations of any potential application and cause the loss of people’s lives and properties. Unfortunately, most of the current solutions cannot effectively detect rogue nodes due to the continuous context change and the inconsideration of dynamic data uncertainty during the identification. Although there are few context-aware solutions proposed for VANET, most of these solutions are data-centric. A vehicle is considered malicious if it shares false or inaccurate messages. Such a rule is fuzzy and not consistently accurate due to the dynamic uncertainty of the vehicular context, which leads to a poor detection rate. To this end, this study proposed a fuzzy-based context-aware detection model to improve the overall detection performance. A fuzzy inference system is constructed to evaluate the vehicles based on their generated information. The output of the proposed fuzzy inference system is used to build a dynamic context reference based on the proposed fuzzy inference system. Vehicles are classified into either honest or rogue nodes based on the deviation of their evaluation scores calculated using the proposed fuzzy inference system from the context reference. Extensive experiments were carried out to evaluate the proposed model. Results show that the proposed model outperforms the state-of-the-art models. It achieves a 7.88% improvement in the overall performance, while a 16.46% improvement is attained for detection rate compared to the state-of-the-art model. The proposed model can be used to evict the rogue nodes, and thus improve the safety and traffic efficiency of crewed or uncrewed vehicles designed for different environments, land, naval, or air.

## 1. Introduction

Road collisions are on the rise and, by 2030, they are anticipated to be the sixth leading cause of death [1,2]. Every year, millions of people die on the roads throughout the world due to traffic accidents, with 40 times as many people suffering injuries [1]. Accidents are also the primary source of traffic congestion, which significantly impacts economic activity [3]. As a result, billions of dollars are wasted because of injury treatments, material loss, lost operating time, and additional fuel consumption [4]. Human mistake is responsible for almost 90% of all accidents [5]. Vehicle automation is among the main aspects of future intelligent transportation systems (ITS) to resolve such problems. Automation can replace (or at least help) human drivers with electronic and mechanical devices to provide both road safety and traffic efficiency [6]. Individual vehicles can autonomously predict traffic anomalies, such as accidents and congestion in real time before the actual happening if instantaneous traffic information of neighboring vehicles is available in each vehicle. As a result, the idea of a vehicular ad hoc network (VANET) arises to enhance road safety and traffic efficiency by providing reliable, up-to-date context data about vehicles in the vicinity [7].

In VANET, with hundreds of sensors and communication technologies, vehicles can exchange real-time data on their state, road conditions, and traffic status [8,9]. Vehicles or infrastructure roadside units (RSUs) analyze neighboring vehicles’ information, autonomously detect traffic anomalies, and change their behaviors accordingly to avoid accidents and congested areas [10,11]. Based on the co-operation concept and the shared observations, a wide range of applications have been suggested for safety, traffic efficiency, entertainment, and commerciality [12]. The availability of continuous and reliable recent vehicle context information, such as position, velocity, and directions, is essential for these applications to function properly [13,14,15,16,17]. Unfortunately, due to dynamic and harsh environments, unreliable communication, and the presence of cyber attackers, mobility information suffers from inaccuracy, incompleteness, and untrustworthiness [18,19,20,21]. The co-operative nature of VANET applications attracts cyber attackers to perform a successful attack. Because vehicles rely on context information for decision-making to preserve network agility and provide safety and traffic efficiency, spreading false information by rogue nodes results in catastrophic failure, including the loss of lives and property and affecting economic sustainability [18].

Security is an essential requirement in VANET, as the attackers can exploit the co-operative nature of VANET applications and inject false information that may cause traffic illusions and trigger vehicles to take wrong but life-critical decisions. Misbehaving vehicles that send bogus information can cause many consequences on network performance, road safety, and traffic efficiency. The presence of misbehaving vehicles can disrupt the deployment of any potential VANET applications, protocols, or services [22,23,24,25,26,27]. Securing VANET using prevention mechanisms is expensive and not enough [27,28]. VANET is also vulnerable to internal attackers in the vehicles’ onboard unit because vehicles work in a hostile environment where the owner can modify, customize, or tamper with communication and computation units. For example, an attacker can trigger the vehicle to send false information about road status, such as slippery roads, by simulating the environment to vehicles sensors. Since preventing rogue vehicles from sending false information cannot prevented, detecting misbehaving vehicles is a critical security requirement for VANET [22,29].

Although various misbehavior detection solutions have been proposed for VANET, detecting misbehaviors still has a research challenge [21]. These solutions can be categorized into two main approaches based on their detection objectives: entity-centric and data-centric [30,31]. The data-centric is used for real-time applications and privacy-protected environments. In contrast, the entity-centric approach is used for long-term detection after enough vehicle data are collected in a centralized location (e.g., the traffic authority center). The performance of the data-centric approach depends on the quality of the information collected from neighboring vehicles. Meanwhile, the performance of the entity-centric depends on the accuracy of the data-centric approach [32,33,34,35]. Unfortunately, due to the harsh vehicle environment and unreliable communication, one cannot guarantee the quality of the information being acquired and shared between vehicles. Therefore, misbehavior detection solutions must be aware of the context. Otherwise, it will generate high false alarms or/and low detection rates depending on the particular vehicular context situation.

Many studies show that context-aware solutions are more practical and effective for VANET [30,32,33,34,35]. However, few context-aware misbehavior detection models were proposed for VANET [32,33,34]. Due to the high dynamic vehicular context, these solutions rely on data-centric classifiers where the mapping between shared uncertain information and vehicle class is fuzzy. Therefore, there is no deterministic correlation between the quality of shared information and the malicious intent of the vehicles. Moreover, machine-learning-based techniques in [33,34,35,36,37] are scenario-specific and assume a stationary correlation between data accuracy and vehicle class, which is not always the case in the highly dynamic context. Accordingly, such an assumption leads to low detection accuracy. To this end, this paper focuses on improving detection performance. More precisely, we intend to answer the following question: how a vehicle can locally detect misbehaving vehicles (rogue nodes), especially in the early attack stage in the highly dynamic, harsh vehicular environment.

This paper proposes a fuzzy-based context-aware misbehavior detection model to detect rogue nodes locally and in their early attack stages. The main aim is to replace the static security thresholds with adaptive context references that are aware of the context to improve the overall detection accuracy. The proposed fuzzy-based context-aware MDS (FCA-MDS) consists of four main phases. Firstly, each vehicle measures the quality of its observation using state-of-the-art acquisition algorithms, such as presented in [24]. Secondly, vehicles evaluate the reliability of the communication by sharing their observations and the quality of these observations using the state-of-the-art adaptive broadcasting scheme presented in [25]. Thirdly, a fuzzy inference system is built to estimate the dynamic context reference. Finally, a data-centric misbehavior detection technique is built to assess the accuracy of incoming context information from nearby vehicles based on their divergence from the dynamic context reference. The divergence of the vehicle’s score from the built context reference using the proposed fuzzy inference system determines whether it is rogue or legitimate. Rogue nodes differ significantly from the context reference. The Next Generation Simulation dataset (NGSIM) [26] was used to evaluate the proposed solution. The vehicles’ trajectories are replayed in a MATLAB simulation environment. A dataset is collected for each vehicle containing neighboring vehicles’ context information with their accuracy and reliability. This study made the following contributions:

1. A fuzzy-based context-aware misbehavior detection model is proposed to effectively detect rogue nodes (misbehaving vehicles) that spread false context information in VANET. Vehicular context is represented by the quality and the reliability of the information created by a set of neighboring vehicles.
2. Due to the high dynamicity of vehicular context, the decision about the maliciousness of vehicles is fuzzy. Thus, a fuzzy inference system is constructed to evaluate the maliciousness of vehicles according to the current context on time.
3. Based on the output of the developed fuzzy inference system, a dynamic context reference is built online. Rogue nodes are the vehicles that significantly diverge from the context reference. This dynamic context reference is more flexible than solely depending on statistical evaluation due to the use of linguistic methods, which is similar to human reasoning.
4. Extensive testing was performed to evaluate and validate the proposed FCA-MDS model. Results of the experiments show that the proposed model outperforms the state-of-the-art models. It attains 83.38% overall performance in terms of F-measure, which is 7.88% higher than the state-of-the-art model.

The rest of this paper is organized as follows. In Section 2, the related works are reviewed with critical analyses. The proposed fuzzy-based context-aware misbehavior detection scheme is presented in Section 3. In Section 4, the performance evaluation and experimental procedure are explained. Results are presented and discussed in Section 5, and in Section 6, the study is concluded.

## 2. Related Works

Misbehavior in terms of sending false information in VANET has been the subject of many studies in recent years. Many misbehavior detection solutions were proposed. These solutions can be classified into three categories: data-centric, entity-centric, or hybrid. The data-centric approach is commonly used in research to detect false information, and thus thwart the misbehaving nodes. In the data-centric approach, messages are evaluated based on the consistency and plausibility of their content data. Meanwhile, in the entity-centric approach, vehicles are evaluated based on their reputation or role. For example, police vehicles are more trusted than users’ vehicles. In the hybrid approach, researchers integrate data-centric and entity-centric approaches into one model. That means the vehicles are evaluated based on the validity of their generated data. Because of the highly dynamic nature of vehicle environments, the shared context information among vehicles becomes unreliable and inaccurate. The unreliability occurs because of losing the messages due to the congestions in the communication channel when the traffic density is high. In contrast, inaccuracy occurs due to the uncertain noise environment where the vehicles move. For example, the positioning accuracy model changes according to time and space. Accordingly, recent solutions for misbehavior detection are based on a context-aware approach, such as the solutions in [30].

Authors in [30] devised a context-aware misbehavior detection model for VANET. An adaptive context reference that considers data uncertainty and unreliability has been constructed online using Kalman and Hampel filter. Kalman filter was used to track the inconsistencies of the sequence of data received from a neighboring vehicle, while the Hampel filter was used to track the special change to detect the abnormal messages. A message is judged false if it deviates significantly from the context reference. However, it is difficult to collaborate the threshold depending solely on data collected from neighboring vehicles. The thresholds that construct the models are calculated online assuming normal distribution, which is not necessary, especially if there are not enough data, such as in the case of low density. Authors in [31] proposed a misbehavior detection model by constructing a classifier using an artificial neural network (ANN). The features were derived from the communication reliability of the nodes and the uncertainty of the data. Although the proposed model shows effectiveness in detecting misbehaving vehicles, the model assumes that the relationship between input features and vehicle class is deterministic, which is not always valid in an ephemeral network, such as VANET. Authors in [32] proposed a misbehavior-aware intrusion detection model for VANET. Each vehicle trains a classifier using random forest (RF) algorithm and shares it with its neighbors; vehicles whose classifiers deviate much from others are considered misbehaving vehicles. However, it is difficult to train different classifiers for each vehicle. Authors in [33] extracted three sets of features related to data consistency, plausibility, and behavioral features. Kalman and Hampel’s filter were used to extract data consistency features, plausibility features were extracted using physical models, such as overlaps of the vehicle’s movement model, and behavioral features were extracted from broadcasting behavior of the vehicles. Three classifiers were constructed and the final decision is taken based on aggregating the output of the three classifiers using a majority voting algorithm. However, such a model relies on parametric statistical representation, which is not suitable for highly stochastic processes due to highly dynamic networks. Authors in [33] improved the model proposed in [32] by extracting features from the parameters of the statistical model and their output score to train an ensemble of classifiers using the random forest (RF) algorithm. However, similar to [33], neither the parameters of the statistical models nor the statistical thresholds are accurate for representing vehicular context. The decision by the classifiers is misloaded by the inaccurate representation. Authors in [38] proposed a misbehavior detection scheme to detect bogus information. However, the proposed scheme is data-centric, which focuses on classifying the messages into true or false based on the consistency and plausibility of the information, as proposed in [32]. Such a solution does not include identifying the rogue node, which is challenging in VANET.

Zhang, Chen [36] proposed a misbehavior detection model using support-vector machine (SVM) and Dempster–Shafer theory (DST). Two trust models were constructed, one for data and the other for the vehicles. A message propagation-based classifier was designed based on the SVM to classify vehicle broadcasting behaviors. DST is used to aggregate the reports made by a trusted authority. However, the dynamic context uncertainty was not considered. Moreover, the model relies on reputation and long-term trust establishment, which is complex and is not suitable for early detection and new misbehaving nodes. Authors in [37] investigated different machine learning techniques to design a misbehavior detection model. Then, an ensemble of two machine learning techniques, namely k-nearest neighbor (kNN) and random forest (RF) classifiers, were used to construct the detection classifier. However, the proposed model is scenario-specific and cannot be generalized. Moreover, the context dynamic uncertainty was not considered while extracting the features for classifications.

To summarize, most of the existing misbehavior detection schemes lack in considering the highly dynamic context of the vehicular network. Most current solutions either rely on static and predefined static thresholds or assume that the mapping between the input features and vehicle class is stationary, which is not always true. There are few context-aware models proposed for VANET. However, many of these solutions are data-centric. That is because the mapping between sharing false or inaccurate information is fuzzy. There is no deterministic correlation between inaccurate information and the malicious intent of the vehicles. To this end, this study proposes a fuzzy-based context-aware misbehavior detection model for VANET. The aim is to improve the detection rate while maintaining low false alarms. A detailed description of the proposed model is presented in the following section.

## 3. The Proposed Fuzzy-Based Context-Aware Approach

**Figure 1:**
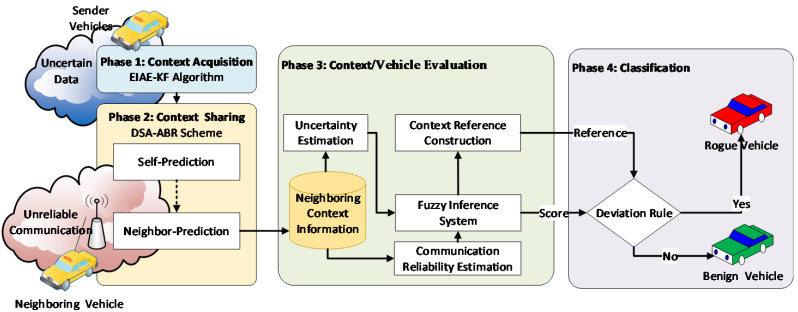
The proposed fuzzy-based context-aware approach for detecting rogue nodes.

As shown in Figure 1, the proposed fuzzy-based context-aware misbehavior detection model (FCA-MDS) consists of four main phases, as follows. The first phase is the context acquisition phase, in which each vehicle is responsible for acquiring its observations from its sensors, as well as filtering the noise. The second phase is the context sharing phase, in which the observations that have been collected by each individual are broadcasted and collected by all neighboring vehicles in the same communication range. The third phase is the evaluation phase, in which the context is evaluated in terms of the uncertainty of the observations and the reliability of the communication. Each vehicle also is evaluated in terms of the uncertainty and reliability of its generated observations and sharing behavior using the fuzzy inference system. The fourth phase is the classification phase, in which vehicles are classified based on their fuzzy-based scores. Vehicles that deviate a lot from the context reference are considered rogue vehicles (or misbehaving vehicles), otherwise they are benign vehicles.

### 3.1. Phase 1: Context Acquisition Phase

Vehicles obtain their mobility information using onboard sensors. However, because of the dynamic and hostile environment, the quality of the collected observations is uncertain. It depends on the context, causing vehicles to process and transmit uncertain information, potentially resulting in disastrous safety and traffic efficiency effects. For this reason, a context acquisition algorithm should be aware of the accuracy of the acquired information. Although many acquisition algorithms have been proposed for VANET, few context acquisition algorithms can estimate the accuracy of the acquired information, such as in [24]. As a result, during the context acquisition phase in this research, the enhanced innovation-based adaptive estimation Kalman filter (EIAE-KF) technique was used. The EIAE-KF produces two vectors as output: context information and the uncertainty of the obtained data.

### 3.2. Phase 2: Context Sharing Phase

As previously stated, vehicles should broadcast the gathered context information from their sensors to the surrounding vehicles. However, due to the dynamic and unique vehicle characteristics, such as density, speed, and environment, the communication channel is not reliable and is context-dependent. The context sharing scheme should be able to cope with such a high dynamic context so that vehicles can share high accurate and reliable context. Although many context sharing schemes have been proposed for VANET, few context-aware schemes can preserve the quality of the information during the sharing phase. The driving-situation-aware adaptive broadcasting rate strategy (DSA-ABR) [25] is one of these techniques employed in this research. DSA-ABR can minimize each vehicles’ broadcast rate based on their driving status while giving up-to-date context information every 100 milliseconds per surrounding vehicle. The context information received from surrounding cars using the DSA-ABR technique results from this step.

In this study, vehicles use the DSA-ABR scheme [25] to broadcast their context information with the vehicles in their vicinities. DSA-ABR scheme comprises two algorithms, one for efficient broadcasting and the second for accurately reconstructing the trajectories of the neighboring vehicles. The broadcasting algorithm works based on the concept of the broadcast if necessary. Only the critical context information is broadcasted. It contains a prediction mechanism that mimics the neighboring vehicles when reconstructing the trajectories of the vehicles using minimum context information. That is, vehicles broadcast the data if there is an unpredictable change by neighboring vehicles. The second algorithm is used to construct the trajectories of the neighboring vehicles using a minimum set of context information. Both algorithms utilize the Kalman filter for estimating the context information and their uncertainties to improve the estimation accuracy.

### 3.3. Phase 3: Context/Vehicle Evaluation Phase

There are two evaluation steps in this phase. The first is to evaluate the context by constructing a context reference. The second is to evaluate the vehicles based on the quality of their generated observations. In the first phase, the context reference is devised using the fuzzy inference system as follows. Two fuzzy variables used the context uncertainty and message arrival rate, which are obtained from the first and second phases, respectively. A detailed explanation of these two variables is presented in the subsequent subsections.

#### 3.3.1. Uncertainty Estimation

The uncertainty of the information of each vehicle is calculated based on the innovation error of the Kalman Filter used in the neighboring predictor algorithm in the previous phase. Let $Q_{k}$ denote the process noise covariance at time epoch k, $R_{k}$ denotes the measurement noise covariance, $P_{k}^{+}$ and $P_{k}^{-}$ denote the posterior and prior estimation error covariance, respectively, F denotes the transmission matrix, and *H* is the mapping matrix between prediction and measurements. Then, according to the Kalman filter algorithm, the prior estimation of uncertainty can be calculated as follows:

(1) $$P_{k}^{-}=FP_{k-1}^{+}F^{T}+\;Q_{k}$$

The Kalman gain $K_{k}$ at time epoch k is calculated as follows:

(2) $$K_{k}=P_{k}^{-}H^{T}C_{k}{}^{-1}$$

where $C_{k}{}^{-1}$ is the inverse matrix measurement uncertainties of $\;C_{k}$ in terms of the innovation error of the Kalman filter $z_{i}$ for time window m. $z_{i}$ denotes the disturbance between measurements and prediction models. The $C_{k}$ can be computed as follows:

(3) $$C_{k}=\frac{1}{m}\overset{k}{\underset{i=k-m+1}{\sum }}z_{i}z_{i}^{T}$$

By calculating the measurement uncertainties, Kalman gain $K_{k}$ is obtained. Kalman gain is used to penalize either the prediction or the measurements model for accurate estimation. However, the noise in the vehicle environment is stochastic highly dynamic, and does not have a predetermined model. Many existing models assume that noise is normally distributed, which leads to inaccurate estimation of the uncertainty and produces inconsistent estimation, which, in turn, increases the false positive rate. Therefore, the autocorrelation test detects whether the noise is normally distributed or correlated noise to calculate the uncertainty using the correct noise model. Accordingly, the autocorrelation function in the following equation is used for the test:

(4) $$\rho_{k}=\frac{\sum_{k=1}^{m-1}\left(z_{k}-\mu_{\varepsilon }\right)\left(z_{k+1}-\mu_{\varepsilon }\right)}{\sum_{k=1}^{m}{\left(z_{k}-\mu_{\varepsilon }\right)}^{2}}\;$$

where $\rho_{k}$ is the autocorrelation of the innovation sequence $z_{k}$ for a period of m epochs. Then, if the absolute value $\left|\rho_{k}\right|$ of the autocorrelation is greater than $\;2/\sqrt{m}$, i.e., $\;\left|\rho_{k}\right|>2/\sqrt{m}$, then, according to the variance sum law statistic of random variables [4], the uncertainty can be calculated using the standard deviation of the prediction model of the Kalman filter using the following equation:

(5) $$\sigma_{DR\left(k\right)}=\sqrt{\sigma_{p_{i}}^{2}+k^{2}\times \sigma_{v}^{2}\;}$$

where $\sigma_{p_{i}}^{2}$ is the variance of the vehicle positions predicted during the Kalman filter prediction phase, while $k^{2}\times \sigma_{v}^{2}$ is the variance of the velocity times the square of the number of time epochs k. If the $\;\left|\rho_{k}\right|\leq \;2/\sqrt{m}$, then the uncertainty can be calculated according to the following equation:

(6) $$\sigma_{AKF}\left(k\right)=\varepsilon_{est\left(k\right)}=(I-K_{k}H)F\varepsilon_{est\left(k-1\right)}+(I-K_{k}H)v_{k-1}-K_{k}w_{k}$$

where $\sigma_{AKF}\left(k\right)$ is the Kalman filter uncertainty when the autocorrelation of the innovation sequence is approaching zero, which is the time where the noise in the vehicle environment follows normal distribution as calculated in [24]. Algorithm 1 shows how each vehicle can calculate the uncertainty of its generated context information.
**Algorithm 1:** Estimate Data Uncertainty of Each vehicle**1: Initialize**$,\;Q_{k-1},\;R_{k-1},P_{k-1}^{+}$,F, *H* **2: FOR** Each Time Epoch k **3:**    Calculate the prediction error covariance            $P_{k}^{-}=FP_{k-1}^{+}F^{T}+\;Q_{k}\;\;\;$ **4:** $\mathrm{Calculate}\;\mathrm{Kalman}\;\mathrm{Gain}\;K_{k}=P_{k}^{-}H^{T}C_{k}{}^{-1}$ **5:** $\mathrm{Compute}\;P_{k}^{+}=\left(I-K_{k}H\;\right)P_{k}^{-}\;$ **6:** Compute the autocorrelation of the innovation sequence            $\rho_{k}=\frac{\sum_{k=1}^{m-1}\left(z_{k}-\mu_{\varepsilon }\right)\left(z_{k+1}-\mu_{\varepsilon }\right)}{\sum_{k=1}^{m}{\left(z_{k}-\mu_{\varepsilon }\right)}^{2}}$ **7: IF**$\;\;\;\left|\rho_{k}\right|>2/\sqrt{m}$ **THEN** **8:**  $\sigma_{DR\left(k\right)}=\sqrt{\sigma_{p_{i}}^{2}+k^{2}\times \sigma_{v}^{2}}$ //Estimation is not optimal **9: ELSE** **10:**  $\sigma_{AKF}\left(k\right)=\varepsilon_{est\left(k\right)}=(I-K_{k}H)F\varepsilon_{est\left(k-1\right)}+(I-K_{k}H)v_{k-1}-K_{k}w_{k}$ **11: CONTINUE LOOP**

#### 3.3.2. Communication Reliability Estimation

The second phase includes estimating the communication reliability in terms of message arriving rate. Because many applications rely on the context information of all neighboring vehicles, sharing context information is essential. However, due to the high mobility of vehicles, the context information streams shared between vehicles are intermittent due to the variety of vehicles’ velocity. Vehicles may go in and out of each other’s communication ranges. Thus, their context information stream is sporadic, which leads to inaccurate information. Therefore, the message arrival rate is calculated by each vehicle for each neighboring vehicle in their vicinities according to the following equation:

(7) $$\mathrm{Message}\;\mathrm{Arrival}\;\mathrm{Rate}=\frac{\sum_{i=1}^{m}\left(\mathrm{arrived}\;\mathrm{messages}\;\mathrm{for}\;\mathrm{each}\;\mathrm{vehicle}\right)}{m}$$

where *m* is the length of the time window in terms of the number of time epochs.

#### 3.3.3. Fuzzy-Based Context Reference and Vehicle Scores

The context reference is built using a fuzzy inference method in this phase. Fuzzy logic is the generalization of crisp logic (Boolean logic), in which a variable’s truth value is represented by a real integer between one and zero. A fuzzy inference system (FIS) is a rule-based system that can automate human decision-making by simulating human thinking. The fuzzy inference system consists of two input variables and one output variable. The inputs are the overall context uncertainty, which is calculated by taking the average of the uncertainties of all neighboring vehicles, as shown in Algorithm 1. The second variable is the average message arrival rate, which is calculated based on Equation (7). The output is the context reference calculated by each vehicle using fuzzy rules. A fuzzy rule is used to map the input variables to calculate the output variable, i.e., the context reference. The proposed FIS in this study is constructed by following the Mamdani fuzzy inference method because it is intuitive and easy to understand and derived based on human expert knowledge. Accordingly, the proposed FIS consists of three steps: fuzzification, inference engine, and defuzzification. The fuzzification step includes the identification of the input variables, the generation of the fuzzy sets, and the selection of the membership function. For example, the traffic flow is low if the vehicles are not freely moving on the road, while it is high if they freely move on the road. Similarly, the uncertainty is high if the noise has no known distribution, it is medium if the noise is dynamic and has a known distribution, and it is low if the noise has a known distribution and can be modeled.

**Figure 2:**
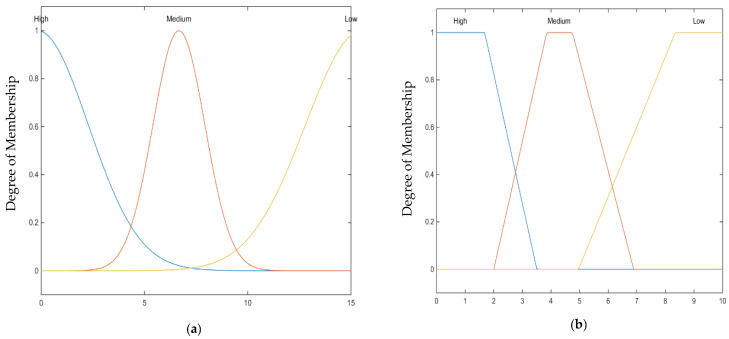

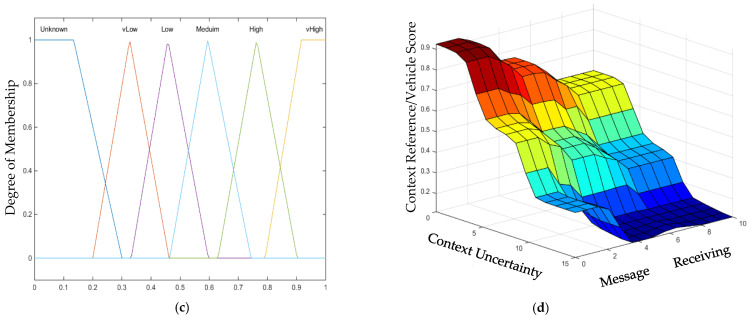
Context reference/vehicle score fuzzy model. (**a**) Context Uncertainty (Innovation Error). (**b**) Message Receiving Rate. (**c**) Context Reference/Vehicle Score. (**d**) Context Reference/Vehicle Score.

The following is how the membership function was determined.The uncertainty of the information collected by the neighboring vehicles has been assumed to have a random normal distribution because they rely on independent sensors. Therefore, the membership function of the context uncertainty variable is the probability density function for the normal distribution. Similarly, the average message arrival rate is used as the second fuzzy input variable. Although the message arrival rate has been proved to have Poisson distribution and, according to the central limit theory, this distribution will end up with normal distribution if the sample size is large, the trapezoidal membership function is used in this study. The intuition behind that is, for real-time applications, the sample size will be very low, and thus the probability distribution will be biased. Consequently, the trapezoidal membership function is used in this study to fuzzify the message arrival rate variable. Thus, the context is represented by the fuzzy output according to the fuzzy input of the average of the uncertainties and message arrival rate values of the neighboring vehicles. Similarly, vehicles are evaluated based on their reported uncertainties and arrival rate. Figure 2a–c shows the membership functions of input and output variables, while Figure 2d shows the mapping between the input variables and output variables.

As shown in Figure 2a, the uncertainty of the context is modeled as three random distribution functions: low, medium, and high. Three fuzzy sets are used to resample the three vehicular context environments: low, medium, and high uncertainty. Low uncertainty happens during vehicle movement in open spaces, while medium uncertainty happens during vehicle movements under trees or in cloudy weather [31,35,36]. Meanwhile, the high uncertainty happens during vehicle movements in upran environments under bridges and skyscrapers or during heavy rain [31,35,36]. Because neighboring vehicles in the same geographical areas share the same context, the use of normal distribution for representation is reasonable. Moreover, the uncertainty is represented using the innovation error of the Kalman filter, which is usually a Gaussian process. The message receiving rate is represented by three fuzzy sets with a trapezoidal membership function. The message arrival rate is proven to have a Poisson distribution that tends to have normal distribution according to the central limits theorems for the long run where enough data samples are collected [39,40]. However, due to the highly dynamic vehicle movements where a small number of vehicles can successfully broadcast the messages, the trapezoidal membership function is more practical to represent the message’s arrival rate. Figure 2c shows the fuzzy output of the proposed fuzzy inference system, which is represented by six fuzzy sets with triangular membership functions. This output represents the current vehicle context score and is used to construct the local context reference. For simplicity, triangular membership functions are selected to represent the output of combining the two members drawn from a normal distribution and trapezoidal membership function.

Moreover, according to the studies [39,40,41], selecting sample shapes, such as triangular, is a reasonable decision for practical applications, as long as there is overlapping between fuzzy sets. As the estimation uncertainty of the Kalman filter increases, the message arrival rate should be increased due to high uncertainties about the data. Thus, a low value approaching zero represents the vehicle score and context reference. Meanwhile, during low estimation uncertainty, the message arrival rate should represent the traffic flow behavior, which will be reflected in the fuzzy output set where vehicle score and context reference varies from 1 to 0.5 to represent such dynamic context. Figure 2d shows the output space of the mapping between the fuzzy input and output variables after applying the fuzzy rules in the proposed inference system.

The second step of constructing the FIS is to construct the inference engine. The inference engine consists of a set of rules called knowledge-based. The knowledge base is built using domain expertise and therefore is employed to express the activity in linguistic form. These rules map the input to the output based on a careful understanding of how the communication reliability in terms of message receiving rate and context uncertainty affect the misbehaving vehicle’s ability to conduct a successful attack. In this study, nine rules were created to build the proposed FIS. For example, if the traffic flow is low and the information uncertainty is high, then it is expected that the attacks might not be very successful; thus, the vehicle will score low malicious (low attack risk). Meanwhile, if the traffic flow is low and uncertainty is low, the attack is likely, then the vehicles will be strictly scored (high risk of the attack). The third step of constructing the FIS is to perform the defuzzification. Because the output of the fuzzy rule is fuzzy, such as very high, high, medium, low, or very low, which is in linguistic form and not suitable for calculations, such output must be defuzzed. This study used the centroid defuzzification approach for defuzzification. The centroid technique works by calculating the output’s center of gravity, which is the aggregate shape of the input variables. The centroid of fuzzy sets is calculated as follows.

(8) $$x_{c\left(j\right)}=\frac{\int_{i=0}^{m}\mu \left(x_{i}\right).x_{i}}{\int_{i=0}^{m}\mu \left(x_{i}\right)}\;\forall \;neigboring\;vehicle\;j\;\in ℵ$$

where $x_{c}$ is the center of the combined output fuzzy shape of vehicle j belonging to the set of neighboring vehicles ℵ, and m is the length of the interval of the region bounded by $I=\left[0,m\right]$ in the x-axis of the output variable. Thus, the context reference parameters are calculated as follows:

(9) $$\mu =\frac{\sum_{i=1}^{n}x_{c\left(i\right)}}{n}$$

(10) $$\sigma =\sqrt{\frac{\sum_{i=1}^{n}{\left(x_{c\left(i\right)}-\mu \right)}^{2}}{N}}$$

where μ and σ denotes the mean and standard deviation of the center of gravity of the output fuzzy set, which represents the proposed dynamic context reference.

### 3.4. Phase 4: Classification Phase

In the classification phase, a vehicle is classified as either genuine or malicious according to the fuzzy-based output of the FIS. A vehicle whose fuzzy score deviated much from the context is considered rogue, otherwise it is genuine. A statistical model with a normal probability distribution assumption has been used in this study to classify the vehicles. Thus, the classification model can be expressed as follows:

(11) $$f\left(x,\;\mu ,\sigma \right)=\left\{\begin{array}{cc} Genuine & \mu -T\sigma \leq x_{c\left(i\right)}\leq \mu +T\sigma \\ Rogue & p\left(x\right)>\mu +T\sigma \;or\;p\left(x\right)<\mu -T\sigma \end{array}\right.$$

where $p\left(x\right)$ is the probability density of the fuzzy output x, μ is the mean of the context reference μ, and σ is the standard deviation of the fuzzy output (the parameters μ and of the context), and T is a threshold that has been heretically selected.

## 4. Performance Evaluation

In this section, the activities that have been used for validating and evaluating the proposed FCA-MDS model are described. The common evaluation procedures used by the state-of-the-art models have been used [5,6]. These activities include dataset collection and preprocessing, environmental noise injection, simulation of message losses, and simulation of the rogue nodes. This study used the Next Generation Simulation (NGSIM) datsdet [26] to evaluate the proposed model. NGSIM includes over 5000 vehicle trajectories and has been used in related research [30,33,34]. Vehicle trajectories have been replayed in the simulation environment, and vehicles have been modeled acquiring and sharing context information with their surroundings. Matlab program has been used to simulate vehicular environmental noises, communication losses, and rogue nodes’ activities.

### 4.1. Datasets’ Source and Preprocessing

In this study, the Next Generation Simulation (NGSIM) [26], which is commonly used in related works to evaluate and validate the MDS models, has also been used to validate the proposed FCA-MDS model. The NGSIM dataset contains ground truth data of more than 5000 vehicles. Many traffic scenarios are presented in datasets relating to drivers’ behavior, vehicle density, velocity, and traffic flows. The dataset includes the ground truth information related to vehicles’ local and global positions (longitude and latitude) sampled every 100 ms. It also includes the velocity (longitude and latitude), acceleration, direction, vehicles’ type, dimensions, and lane number. For prepossessing, missing data and outliers were replaced by averaging and smoothing the values. Following the findings of Thiemann et al. [42], the velocity measurements were smoothed using the exponentially weighted moving average method (sEWMA). The derivative of velocity over time was used to estimate the acceleration. Furthermore, the heading angle was calculated by taking the derivative of position displacement in one axis over displacement in the other.

### 4.2. Simulation of Environmental Noises

The vehicle’s trajectories were subjected to three different forms of noise, including static white noises, dynamic white noises, and dynamic correlated noises. The static and dynamic white noise follows the normal distribution with zero mean, with fixed variance for static noises and time-varying variance for dynamic noises. Meanwhile, the correlated noise is modeled as a random walk process, such as in [30,33,34,43]. Static white noise occurs in the open sky environment such as in driving in the rural environment, such as the highway in the desert; the dynamic noises were reported under trees and cloudy environment; and correlated noises were reported beside skyscrapers, under bridges, tunnels, or earth features [30,33,34,44]. Table 1 shows the three models used to simulate the environmental noise in this study. These models were adopted from our previous experiments in [25], which were used to evaluate the acquisition algorithm EIAE-KF that is used in the first phase of the proposed FCA-MDS model in this study.

**Figure 3:**
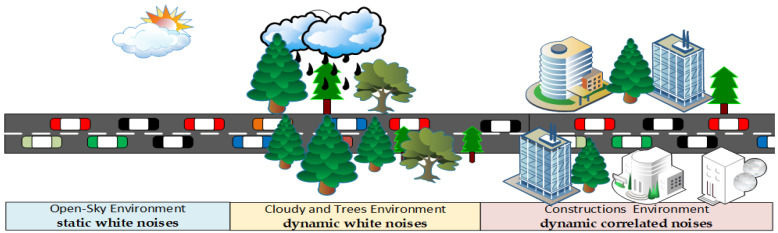
Road noise scenario.

Realistic environmental noises are simulated in this study. The road scenario was divided into three segments, as shown in Figure 3. In each segment, a different noise model was injected into the vehicles located in that segment, as shown in Table 1.

### 4.3. Simulation of Communication Losses

**Figure 4:**
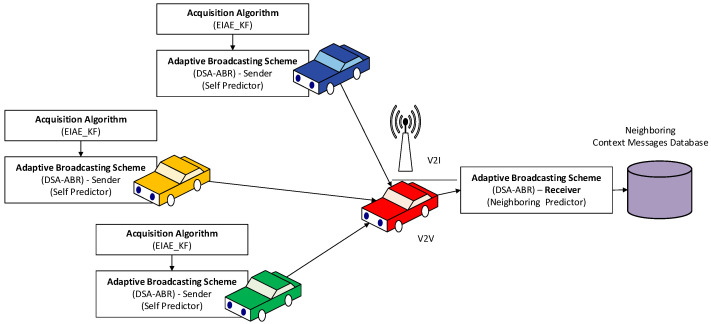
Dataset collection from neighboring vehicles.

Because vehicles move in highly dynamic environments, communication loss is a common problem due to many parameters, including traffic density, vehicle velocity, and obstacles. Due to different vehicles’ speeds and behavior, vehicles go in and out of their communication ranges, which makes messages lost. The communication losses increase when the density of the vehicles increases. Due to the highly dynamic context of VANET, many safety applications require vehicles to share their data every 100 ms (10 messages per second). Such requirements cause channel congestions, and thus communication loss. However, many broadcasting schemes are unreliable for such applications due to the lack of consideration of information accuracy as the main performance measure. As mentioned earlier, the DSA-ABR [3] has been used in this study due to the consideration of dynamic context uncertainty for broadcasting decisions. In DSA-ABR, each vehicle estimates its context information using the Kalman filter every 100 ms. Vehicles also carry out self-prediction of their previous broadcasted information, and, according to the prediction error, it decides whether to send or omit their information. Thus, to simulate communication loss in this study, as suggested by Knuth [8] and proved by Mcquighan [9], the message arrival time in each neighboring vehicle can be modeled as a random Poisson distribution as follows:

(12) $$\mathrm{Next}\;\mathrm{Time}\;\mathrm{Arrival}=\mathrm{Previous}\;\mathrm{Time}\;\mathrm{Arrival}+\frac{-\ln\left(1-u\right)}{\lambda }$$

where u denotes a random value between 0 and 1: $U∼Uniform\left(0,1\right)$, and λ is the actual average arriving rate in each particular time interval (e.g., 1 message per 100 ms). The random Many VANET researchers have employed the Poisson distribution to model message arrival rates [10,11,12]. Figure 4 is adopted from our previous publication in [40] to demonstrate the dataset collected by each vehicle to be used for misbehavior detection.

In this study, nine communication context were simulated using Matlab-based simulator, which was implemented and used in our previous studies [31,34,35]. The IEEE 802.11p/WAVE standards were implemented according to the studies [45,46,47]. Table 2 lists the simulation parameters used in this study. In each communication scenario, different message arrival probabilities were simulated ranging from 1 to 0.01, namely $\lambda =\left\{1,\;0.5,\;0.3,\;0.2,\;0.1,\;0.05,\;0.03,\;0.02,\;\mathrm{and}\;0.01\right\}$. For example, in the ideal scenario, the probability is set to one, so that all broadcasted messages arrive, whereas, for probability equals 0.01 (worst communication scenario), only 1% of the broadcasted messages arrive. Each communication scenario represents a different context scenario in terms of traffic flow situation, including vehicle speeds and density.

### 4.4. Rogue Nodes Simulation

To validate the proposed model in this study, rogue nodes that misbehave by sending false context information were simulated. Due to the absence of a labeled dataset in VANET, researchers in this domain simulate the vehicle’s actions against the false information [13,14]. False information attacks, which can seriously impact road safety, traffic efficiency, and people’s lives, are simulated in this study. Attackers can launch different types of false information, ranging from basic to sophisticated attacks, including sudden or random continuous position jumping, Sybil attack, inaccurate movement patterns, and consistency attacks. The most challenging attack is the consistency attack, in which the attacker tries to generate consistent but fake vehicle trajectories to cause traffic illusions that degrade applications and network performance. These attacks were simulated based on work found in [13,14,33,34,35].

This study randomly selected 10% of vehicles from the NGSIM dataset as rogue nodes to simulate the consistency attacks. Three types of illusion attacks based on incremental positioning jumping were included: creating fake trajectories, copying the history of the trajectories of some neighboring vehicles, and false maneuvering patterns, such as fake breaking and fake lane changing. Such types of attacks are easy to create but difficult to detect. In the simulation environment, each rogue node (misbehaving vehicle) randomly selects one attack type to create fake but consistent trajectories considering the neighboring vehicles’ locations and speeds to avoid overlapping and position jumbling. Misbehaving vehicles tried to report false context inaccurate information regarding their position, speed, direction, or lane.

### 4.5. Expermintal Procedures

For the experiments in this study, 1725 vehicles were used. Nine context scenarios were used in the experiments. In each context scenario, three types of noises were injected as explained in Section 4.2 to represent context uncertainty and one communication scenario as explained in Section 4.3 to represent communication status resulting from the traffic flow situation. For example, if λ=1, then this is ideal communication where no message loss is present, while, if λ=0.01, it represents the worst communication scenario where loss of messages is high due to vehicle density and mobility.

According to the dataset timestamps, in every 100 ms, vehicles’ trajectories were replayed in the Matlab simulation environment. The corresponding noises model was used to inject noises into the vehicles’ trajectories according to their position in the road segment in each time epoch, as shown in Figure 3. A total of 10% of vehicles were randomly selected as rogue nodes. A misbehaving vehicle creates fake but consistent trajectories considering the positions and speeds of the neighboring vehicles to avoid overlapping and position jumbling, and create a more sophisticated attack. These fake trajectories are injected into the datasets and the vehicles are labeled as benign and rogue vehicles. As there are nine communication scenarios, this procedure is repeated nine times.

According to the dataset timestamps, each vehicle uses the EIAE-KF algorithm [24] to estimate its correct context information, such as vehicle position, speed, direction, and acceleration. Accordingly, each vehicle forms the co-operative awareness message (CAMs) and uses the DSA-ABF [25] broadcasting scheme to broadcast the messages to their neighboring vehicles within a 1 km communication range. Each vehicle creates a database to store the received context information from their neighboring vehicles according to the simulated communication scenario. Each vehicle also stores the innovation errors of the Kalman filter to be used for uncertainty estimation for each neighboring vehicle in that particular time epoch, as explained in Section 3.3.1. Then, each vehicle calculates the message arrival rate for each neighboring vehicle, as described in Section 3.3.2. The estimated uncertainty and the message arrival rate are fuzzified using the input fuzzy sets, as explained in Section 3.3.3. Each vehicle invokes the particular fuzzy rules from the knowledge base based on the fuzzified inputs to generate the output fuzzy value. Then, the output fuzzy value is defuzzied using Equation (8) to represent the vehicle score, and the context reference parameters are computed according to Equations (9) and (10). Then, using Equation (11), vehicles are classified into benign or rogue. The results are extracted from the detection reports of 16 benign vehicles that were randomly selected from the dataset to evaluate the proposed FCA-MDS.

### 4.6. Performance Measures

To validate the efficacy of the proposed FCA-MDS model, the accuracy, the detection rate (DR), the false positive rate (FPR), the precession, and the F-measure were used in this study, as they are commonly used measures for the evaluation in the related works [5,43]. Because the percentage of misbehaving vehicles is very low compared with normal vehicles, the overall accuracy performance in this study has been measured using F-measure. F-measure is considered a suitable evaluation metric by many related works because it does not take the true negative into account [30,33,34,43]. In addition, using fixed thresholds, it is easy to either optimize precession or the detection rate (recall). Thus, these two evaluation metrics must be studied together to evaluate the effectiveness of the proposed detection scheme. Thus, F-measure is adopted as the main performance measure to evaluate the proposed model. The following equations are used for calculating the used performance measures in this study:

(13) $$\mathrm{Accuracy}=\frac{TP+TN}{TP+TN+FP+FN}$$

(14) $$\mathrm{FPR}=\frac{FP}{TP+FN}$$

(15) $$\mathrm{DR}\;\left(\mathrm{Recall}\right)=\frac{TP}{TP+FN}$$

(16) $$\mathrm{Precision}=\frac{TP}{TP+FP}$$

(17) $$F-\mathrm{measure}=\frac{2\times \mathrm{Precision}\;\times \mathrm{Recall}}{\mathrm{Precision}+\mathrm{Recall}}$$

### 4.7. Performance Comparison

For the performance evaluation, the findings of the proposed FCA-MDS model have been compared against state-of-the-art models, namely the baseline model as implemented in [38,43], ECT-MDS model [6], and CA-EC-MDS [30,33]. The baseline and ECT-MDS models are non-context-aware, while CA-EC-MDS is a context-aware model. CA-EC-MDS is originally developed as a data-centric model in [30] and converted to modified as an entity-centric model in [33]. The obtained performance has been measured using the aforementioned metrics, namely the accuracy, FPR, DR, and F-measure. The baseline MDS is configured with a fixed threshold set to 1.8. This threshold has been selected because it gives the best performance in terms of F-measure.

## 5. Results and Discussion

**Figure 5:**
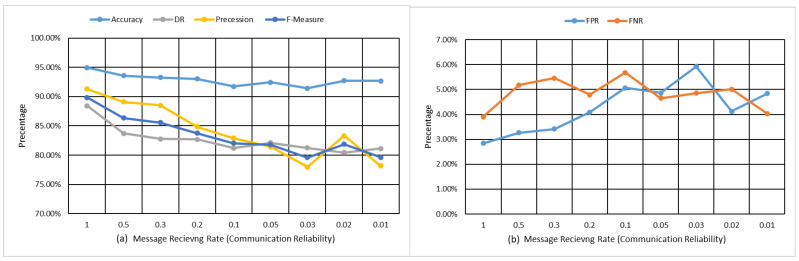
Performance evaluation of the proposed FCA-MDS model in terms of (**a**) accuracy, DR, precession, and F-measure and (**b**) FPR and FNR.

**Figure 6:**
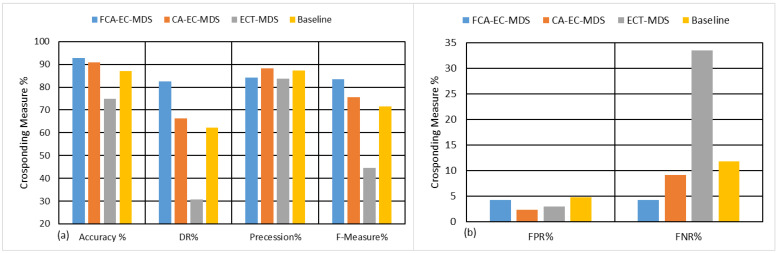
Performance comparison between the proposed FCA-MDS model and the related works in terms of (**a**) accuracy, DR, precession, and F-measure and (**b**) FPR and FNR.

**Figure 7:**
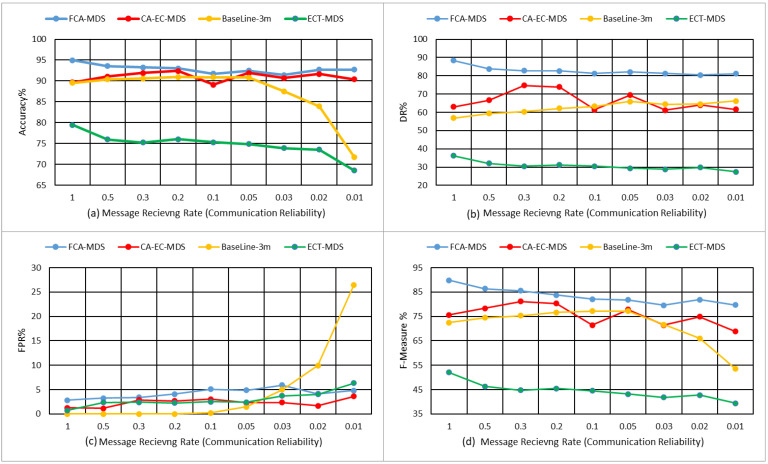
Performance evaluation of the proposed FCA-MDS model in terms of (**a**) accuracy, (**b**) DR, (**c**) FPR, and (**d**) F-measure.

The results of the proposed FCA-MDS model is presented and discussed in this section. Figure 5a,b shows the results obtained by implementing the proposed model, while Figure 6 and Figure 7, as well as Table 3, show the results of the comparisons with the related state-of-the-art models. Figure 5a illustrates the performance in terms of accuracy, detection rate (DR), precession, and F-measure, while Figure 7b displays the false positive rate (FPR) and false negative rate (FNR). The x-axis of Figure 7a,b consists of the nine simulated VANET contexts. In reality, these scenarios represent the communication reliability of the VANET context in terms of the number of received messages due to changes in traffic flows because of the variations in the vehicle’s density and speeds. Each line in Figure 5a,b represents the behavioral performance of the proposed FCA-MDS concerning communication reliability.

As displayed in Figure 5a the accuracy performance slightly degrades when the communication reliability decreases. The proposed model achieves 94.96% accuracy when the communication is optimal (no message loss), while it degrades to 92.69% in the worst studied communication scenario where the message receiving ratio is around 1% of the generated messages. In terms of detection rate, procession, and F-measure, the proposed model achieves 88.42%, 91.30%, and 89.84%, respectively, when the communication is optimal (no message loss), while it degrades in the worst studied scenario to 81.13%, 78.18%, and 79.63% in the detection rate, procession, and F-measure, respectively. This slight degradation is normal because, in high traffic flows and challenging vehicular context, the message receiving rate decreases, and thus the data uncertainty increases and, accordingly, it becomes challenging to differentiate between the benign and rogue vehicles. However, the degradation of the accuracy should not lead to a low detection rate or high classification error. The degradation of the performance in terms of DR, precession, and F-measure increases compared with the accuracy performance. The reason for this degradation is that the number of misbehavior messages generated by a vehicle is less than the number of benign messages. In this case, the F-measure can best describe the performance of the proposed MDS. The classification errors in terms of FPR and FNR, as shown in Figure 5b, slightly fluctuate between 3% and 6%, which indicates the robustness of the proposed model in a highly dynamic context.

To evaluate the performance of the proposed model, Table 3 and Figure 6 show the performance evaluation of the proposed model compared to the related works in terms of average performance. On average, the proposed model archives 92.88% accuracy, 82.65% detection rate, 84.18% precession, and 83.38% F-measure compared to the context-aware approach CA-EC-MDS in [32], which achieves 90.98% accuracy, 66.18% detection rate, 88.18% precession, and 75.5% F-measure. The non-context-aware models achieve lower performance than the context-aware models and fail to strike the balance between precession and recall. The proposed model outperforms the other studied models with most of the performance measures. Although the CA-EC-MDS achieves a better reduction in the false alarms (FPR) compared to the proposed model, its detection rate is 66.18%, which is lower than that achieved by the proposed model (82.65%). In addition, the CA-EC-MDS achieves better precision (88.18%) than the proposed FCA-MDS model (84.18%). However, such achievement is in the favor of increasing the false negative rate (FNR). That is, it fails to strike the balance between precession and recall. Overall, the proposed model outperforms all the other studied models. The proposed FCA-MDS model achieves an 83.38% F-measure compared with 75.5% for the CA-EC-MDS, 44.49% for the ECT-MDS model, and 71.6% for the baseline model. Table 3 also shows that the proposed model is more stable than the other studied models for most performance measures. The accuracy slightly changed (∓0.98%) and ∓3.17% for the overall performance in terms of F-measure.

Figure 7 illustrates the detailed comparison in terms of the robustness of the studied MDS models with context dynamicity. In Figure 7a–d, the x-axis represents nine different context scenarios; in each, the traffic flows of vehicles increases, causing different communication reliability levels. Meanwhile, the y-axis in Figure 7a–d represents the corresponding studied performance measures, namely the accuracy, detection rate, false alarms, and F-measure, respectively.

As can be noticed in Figure 7a the accuracy of the proposed model is stable, with slight degradation when the communication reliability drops. the accuracy of the proposed model remains the highest and more stable than the other studied models. It can also be noticed that both the context-aware model of the proposed FCA-MDS and the CA-EC-MDS are more stable than the non-context-aware model. In terms of detection rate (see Figure 7b), the proposed model outperforms the other studied models; the detection rate remains higher than 80% in all studied scenarios. In terms of false alarm rate (see Figure 7c), the baseline is more stable in the reliable communication scenarios, while it increased rapidly once the communication reliability decreases. The other studied models are more stable and attain a false positive rate lower than 6% in most scenarios. The overall performance in terms of the F-measure (see Figure 7d) shows that the proposed model is the most stable among the compared model. The performance of the CA-EC-MDS fluctuates randomly while the overall performance baseline model drops when the communication channel becomes unreliable. Meanwhile, the overall performance of the ECT-MDS model remains stable at under 55% which is not suitable for VANET’s highly dynamic context. To ensure the statistical significance of the results, the Student test (*t*-test paired with two samples for means) is conducted between the results obtained by the proposed model and the other studied models. Table 4 lists the results for the *t*-test at a 95% significance level.

As presented in Table 4, the results show that there are statistical differences between the proposed FCA MDS and the other related works. As long as the p-value is smaller than alpha (α=0.05), the improvements by the proposed FCA MDS are significant. The t-value represents the level of improvement in the overall accuracy.

To summarize, the proposed fuzzy-based context-aware MDS model (FCA-MDS) outperformed other studied models in terms of overall performance. In general, due to the use of dynamic context reference in the context-aware models and static reference in the non-context-aware models, context-aware models perform better than non-context-aware models. The proposed fuzzy-based context-aware approach has promising results and provides data integrity and a more secure environment for ephemeral networks like VANET, FANET, and drone technology. However, the results showed that there is still room for improvement. One potential improvement could be the use of artificial intelligence techniques to adapt the detection thresholds according to the context. More features should be included for obtaining an accurate representation of the vehicular context.

## 6. Conclusions

The performance of many essential VANET applications needs precise context information about the vehicles in the vicinity. On the other hand, rogue vehicles disrupt the potential of VANET applications by spreading misleading context information, endangering people’s lives and property. Detecting misbehaving vehicles in VANET is a challenging task. Many solutions have been proposed for detecting misbehaving vehicles. However, most of these solutions are data-centric, which maps false data to misbehaving vehicles, which are not always true to the high uncertainty of the context information. That is, vehicles may send false information unintentionally, which increases the false positive rate. Many of these solutions use predefined and static thresholds for detection, which does not hold for dynamic and uncertain contexts. Rogue vehicles misbehave by sending consistent but false information to bypass such predefined detection thresholds. The detection of misbehaving vehicles is fuzzier than the detection of false information messages. In this paper, both vehicular context and vehicles’ behavior are represented by fuzzy variables. Thus, a fuzzy inference system is constructed and used to evaluate both the context and vehicles’ behavior. A context-aware misbehvior detection scheme based on fuzzy logic approach is proposed. Firstly, the features that represent the context are extracted from context information shared by neighboring vehicles. Then, a fuzzy inference system is constructed to evaluate the context and vehicles’ behaviors. A score is generated for each vehicle and the context. Finally, a statistically based classifier is applied to the output of the fuzzy inference system such that vehicles whose scores are close to context score are considered normal. Meanwhile, vehicles that deviate much from the context reference are considered rogue (misbehaving) vehicles. The results presented in this study are promising in terms of the performance achieved by the fuzzy-based context-aware detection approach. However, the use of a predefined detection threshold leads to imprecise detection and increases false alarms. This issue will be addressed in our future work. Moreover, we are planning to incorporate artificial intelligence techniques, i.e., machine learning, to adapt the detection threshold according to a given vehicular context. In addition, more representative context features will be derived for accurate representation.

## Figures and Tables

**Table 1:** The used noise models.

Noise Type	Noise Model	Description
Static Gaussian Noise	$N\left(\mu ,\;\sigma^{2}\right),\;\mu =0,\;\sigma =10m$	Static noise is represented as a normal distribution with mean zero mean (μ=0) and 10 m standard deviation (σ=10m)
Dynamic Gaussian Noise	$N\left(\mu ,\;\sigma^{2}\right),\;\mu =0,\;\sigma =20\;rand()\;m$	Dynamic noise is represented as a normal distribution with mean zero mean (μ=0) and random standard deviation (σ=20 rand() )
Dynamic Correlated Noise	$e_{t}=\alpha e_{t-1}+u$	$\mathrm{Where}\;e_{t}\;\mathrm{the}\;\mathrm{noise}\;\mathrm{at}\;\mathrm{time}\;t\;\mathrm{and}\;\mathrm{represented}\;\mathrm{as}\;a\;\mathrm{random}\;\mathrm{walking}\;\mathrm{process},\;\alpha \;\mathrm{is}\;a\;\mathrm{coefficient}\;\mathrm{to}\;\mathrm{weight}\;\mathrm{previous}\;\mathrm{noise}\;\mathrm{value}\;(\alpha =1),\;\mathrm{and}\;u$ is white noise to represent the harsh environment.

**Table 2:** Simulation parameters.

Simulation Parameter	Configured Value
Communication Protocol	IEEE 802.11p/WAVE
Communication Range	1000 m
Message Generation Rate	10 Hz
Max Broadcasting Rate	10 messages/second
Data Payload	500 Byte
Data Rate	3 Mbps
Propagation Model	Two-ray path-loss
Message arrival probabilities	1 to 0.01
Contention Mechanism	CSMA/CA
Number of Vehicles	1725
Vehicle Speeds	40–100 km/h
Simulation Time	15 min

**Table 3:** Results of performance evaluation.

Model		Accuracy%	FPR%	FNR%	DR%	Precession%	F-Measure%
FCA-EC-MDS (the proposed)	Average	92.88	4.27	4.27	82.65	84.18	83.38
	Deviation	∓0.98	∓0.94	∓0.56	∓2.26	∓4.45	∓3.17
CA-EC-MDS [32]	Average	90.98	2.33	9.15	66.18	88.18	75.50
	Deviation	∓1.05	∓0.78	∓5.03	∓4.77	∓4.03	∓1.05
ECT-MDS [6]	Average	74.79	2.98	33.49	30.65	83.83	44.49
	Deviation	∓2.71	∓1.49	∓3.62	∓2.32	∓6.56	∓3.32
Baseline [43]	Average	87.037	4.79	11.88	62.25	87.25	71.6
	Deviation	∓5.94	∓8.29	∓0.91	∓3.0	∓0.18	∓7.21

**Table 4:** The statistically significant of the overall performance (F-measure).

Tested Models	*t*-Value	*p*-Value	Significance
CA-EC-MDS [32]	5.63845577	0.000107936	Statistically significant
ECT-MDS [6]	9.91473704	8.59619 × 10^−7^	Statistically significant
Baseline [43]	4.36128591	0.000709148	Statistically significant

## Data Availability

The Next Generation Simulation (NGSIM) dataset that was used in this study is publicly available online at the following link: https://ops.fhwa.dot.gov/trafficanalysistools/ngsim.htm, and can be downloaded directly from the following link: https://data.transportation.gov/Automobiles/Next-Generation-Simulation-NGSIM-Vehicle-Trajector/8ect-6jqj (accessed on 21 February 2022).
